# Efficacy of Neuromuscular Electrical Stimulation in Patients on Hemodialysis: An Updated Systematic Review and Meta-analysis

**DOI:** 10.1016/j.xkme.2026.101368

**Published:** 2026-04-16

**Authors:** Yuhei Otobe, Naoto Usui, Sho Kojima, Nobuyuki Shirai, Yuta Suzuki, Kenichi Kono, Hiroki Yabe, Shota Matsufuji, Tadashi Sofue, Naohiko Fujii, Ichiei Narita, Kunihiro Yamagata, Junichi Hoshino, Ryota Matsuzawa, Masakazu Saitoh

**Affiliations:** 1 Graduate School of Rehabilitation Science, Osaka Metropolitan University, Osaka, Japan; 2 Department of Rehabilitation, Kisen Hospital, Tokyo, Japan; 3 Department of Rehabilitation, Niigata Rinko Hospital, Niigata, Japan; 4 Center for Outcomes Research and Economic Evaluation for Health, National Institute of Public Health, Saitama, Japan; 5 Department of Physical Therapy, International University of Health and Welfare School of Health Science at Fukuoka, Okawa, Japan; 6 Department of Physical Therapy, School of Rehabilitation Sciences, Seirei Christopher University, Shizuoka, Japan; 7 Department of Rehabilitation, Aijinkai Rehabilitation Hospital, Osaka, Japan; 8 Department of Cardiorenal and Cerebrovascular Medicine, Kagawa University, Kagawa, Japan; 9 Department of Nephrology, Hyogo Prefectural Nishinomiya Hospital, Nishinomiya, Japan; 10 Niigata Institute for Health and Sports Medicine, Niigata Sports Association, Niigata, Japan; 11 Department of Nephrology, Faculty of Medicine, University of Tsukuba, Tsukuba, Japan; 12 Division of Preventive and Sports Nephrology, Graduate School of Comprehensive Human Sciences, University of Tsukuba, Tsukuba, Japan; 13 Department of Nephrology, Tokyo Women's Medical University, Tokyo, Japan; 14 Department of Physical Therapy, School of Rehabilitation, Hyogo Medical University, Hyogo, Japan; 15 Department of Physical Therapy, Faculty of Health Science, Juntendo University, Tokyo, Japan

**Keywords:** Hemodialysis, kidney disease, neuromuscular electrical stimulation, meta-analysis, randomized controlled trial, systematic review

## Abstract

**Rationale & Objective:**

Kidney disease is associated with reduced physical function, muscle mass, and quality of life (QoL). Neuromuscular electrical stimulation (NMES) is a promising intervention to address these outcomes. This study aimed to update previous meta-analyses by including the latest randomized controlled trials (RCTs).

**Study Design:**

Systematic review and meta-analysis of RCTs.

**Setting & Study Populations:**

RCTs evaluating effects of NMES in adult patients with kidney disease.

**Selection Criteria for Studies:**

Outcomes included muscle strength, muscle mass, functional capacity, physical performance, activities of daily living, physical activity, health-related QoL, and adverse events.

**Data Extraction:**

A systematic search was conducted in PubMed, Cochrane Library, and ICHUSHI (April 27, 2024).

**Analytical Approach:**

Random-effects meta-analyses were performed using Review Manager 5.4. Risk of bias and certainty of evidence were assessed using RoB 2.0 and GRADE.

**Results:**

Twelve RCTs involving 289 participants were included. All participants underwent hemodialysis; no studies included patients on peritoneal dialysis, with predialysis chronic kidney disease, or post-kidney transplant recipients. NMES significantly improved lower-limb muscle strength (standardized mean difference, 0.84; 95% CI, 0.56-1.11; *P* < 0.001), 6-minute walk distance (mean difference, 48.15; 95% CI, 15.86-80.44; *P* = 0.003), and sit-to-stand performance (standardized mean difference, 0.80; 95% CI, 0.17-1.43; *P* = 0.01), without significantly increasing adverse events. No significant effects were found on appendicular skeletal muscle mass, timed up and go test, Short Physical Performance Battery, activities of daily living, physical activity, or HRQoL. The certainty of evidence ranged from very low to moderate across outcomes.

**Limitations:**

Most studies had small sample sizes and high risk of bias.

**Conclusions:**

NMES is a safe and effective intradialytic intervention for improving lower-limb muscle strength and functional capacity in patients undergoing hemodialysis. High-quality RCTs involving broader kidney disease populations are warranted to further establish its clinical utility.

## Plain-Language Summary

People with kidney disease sometimes become weak and are less able to walk; many cannot exercise enough. We asked whether neuromuscular electrical stimulation (NMES)—electrical pulses delivered through pads or belts to the skin to make leg muscles contract—could help when used during dialysis. We searched PubMed, Cochrane Library, and ICHUSHI and pooled the results of 12 randomized trials (289 participants). NMES increased lower-limb strength and improved walking distance and sit-to-stand performance. It did not clearly improve muscle mass, physical performance scores, daily activities, physical activity, or quality of life. Adverse events were rare and similar to those experienced during usual care. Most trials were small and at risk of bias; therefore, stronger studies are needed. NMES might be a practical and safe add-on in patients who opt for exercise.

Kidney disease is a significant global health concern, affecting approximately 850 million people,^1^^,^^2^ with a global prevalence of >10% of the population.^3^ Patients with kidney disease, including those undergoing dialysis, experience substantial declines in physical function, muscle atrophy, functional capacity, and quality of life (QoL).^4^, ^5^, ^6^ Moreover, these functional impairments contribute to adverse outcomes.^7^, ^8^, ^9^, ^10^ Therefore, effective interventions are essential for improving physical function and overall patient well-being.

Neuromuscular electrical stimulation (NMES) is a promising intervention for enhancing physical function in patients with kidney disease. NMES is a well-tolerated rehabilitation technique that increases muscle mass and strength. Using a portable stimulator, the device delivers low-level electrical impulses through adhesive-pad or belt-type electrodes placed on the skin to activate muscles and mimic the effects of exercise, typically for 20-60 minutes per session. NMES has also been evaluated in populations with limited ability to perform voluntary exercise, such as patients with chronic obstructive pulmonary disease,^11^ chronic heart failure,^12^ stroke,^13^ and critically ill patients in intensive care units,^14^ with potential benefits reported across multiple outcomes.

Two meta-analyses published in 2020^15^^,^^16^ demonstrated that NMES improved muscle strength and functional capacity in patients undergoing hemodialysis (HD). However, these meta-analyses^15^^,^^16^ included randomized controlled trials (RCTs) and nonrandomized studies, potentially introducing a higher risk of bias. Given the accumulation of new RCTs over the past 5 years, an updated meta-analysis is warranted. Furthermore, the present review evaluated a broader set of outcomes, including physical performance, activities of daily living (ADLs), and physical activity, in addition to muscle strength, muscle mass, functional capacity, health-related QoL (HRQoL), and adverse events. In this systematic review and meta-analysis, we aimed to clarify the effects of NMES in patients with kidney disease by updating previous meta-analyses and including the latest RCTs.

## Methods

The study protocol was registered in PROSPERO (CRD42024530961). This systematic review and meta-analysis was conducted in accordance with the Preferred Reporting Items for Systematic Reviews and Meta-Analysis 2020 checklist.^17^

### Ethics Statement

Ethical approval and informed consent were not required because this systematic review and meta-analysis used only published data and did not involve individual participant data.

### Search Strategy

We systematically searched for previously published studies using the following publicly available databases: PubMed, Cochrane Library, and ICHUSHI. A literature search was conducted on April 27, 2024. The detailed search strategies are provided in the Supplementary Material (Table S1).

### Eligibility Criteria

Two independent reviewers conducted the initial screening based on the titles and/or abstracts and performed the second screening, which included full-text reviews. Any disagreements were resolved through discussion with a third reviewer, if necessary.

The inclusion criteria were studies: (1) evaluating the effect of NMES interventions in adults (aged ≥18 years); (2) including a population with chronic kidney disease (CKD), including patients on dialysis or predialysis and kidney transplant recipients; (3) using an RCT design; (4) assessing outcomes related to physical function, functional capacity, physical activity, ADLs, QoL, or adverse events (eg, knee extension strength, 6-minute walk distance [6MWD], Short Physical Performance Battery [SPPB], skeletal muscle mass, HRQoL); and (5) published in English or Japanese. The exclusion criteria were studies: (1) with inappropriate article types (eg, case reports, editorials, and review articles); (2) using study designs other than RCTs; (3) comprising patients with clinically recognized severe cognitive impairment; and (4) comprising patients hospitalized within 3 months prior to enrollment.

### Data Extraction

Two reviewers independently extracted data from the included studies using a unified table and ensured their accuracy. The extracted data included publication details (authors, publication year, country), participant characteristics (sample size, sex, treatment status, mean or median age), intervention details (types of NMES, NMES intervention protocols), and outcome measures. Any discrepancies in the data extraction were resolved through discussion with a third reviewer.

### Risk of Bias and Certainty of Evidence

The risk of bias for all studies included in the meta-analysis was assessed by the Cochrane Risk of Bias tool for randomized trials (RoB 2.0).^18^ Two reviewers independently conducted the assessment and classified the overall risk of bias for each study as low, high, or “some concerns.” The overall certainty of the evidence was evaluated using the Grading of Recommendations Assessment, Development, and Evaluation (GRADE) system^19^ and categorized into 4 levels: high, moderate, low, and very low. Disagreements between the 2 reviewers about the risk of bias or the GRADE evaluation were resolved through discussion with a third reviewer.

### Data Synthesis

Statistical analyses were performed using Review Manager 5.4 (Cochrane Collaboration).^20^ Regarding continuous outcomes, effect sizes were calculated as mean differences (MDs) or standardized MDs (SMDs), each presented with 95% confidence intervals (CIs) depending on the measurement scale used in the included studies. Postintervention means and standard deviations were used for estimating pooled effect sizes. When the standard error of the mean was reported instead of standard deviation, it was converted accordingly. The MD was applied when all studies measured the outcome by the same scale, whereas the SMD was used when different measurement scales were used across studies. Considering dichotomous outcomes, including adverse events, the effect size is expressed as the risk ratio with 95% CI. Forest plots were used for visually representing the meta-analysis results. Heterogeneity among the included studies was assessed by the *I*^2^ statistic, with thresholds of 25%, 50%, and 75% representing low, moderate, and high heterogeneity, respectively.^21^ A random-effects model was used for accounting for variability across the studies.^21^ Publication bias was not assessed by funnel plots because <10 studies were included in the meta-analysis of each outcome.^22^

## Results

### Study Selection

**Figure 1:**
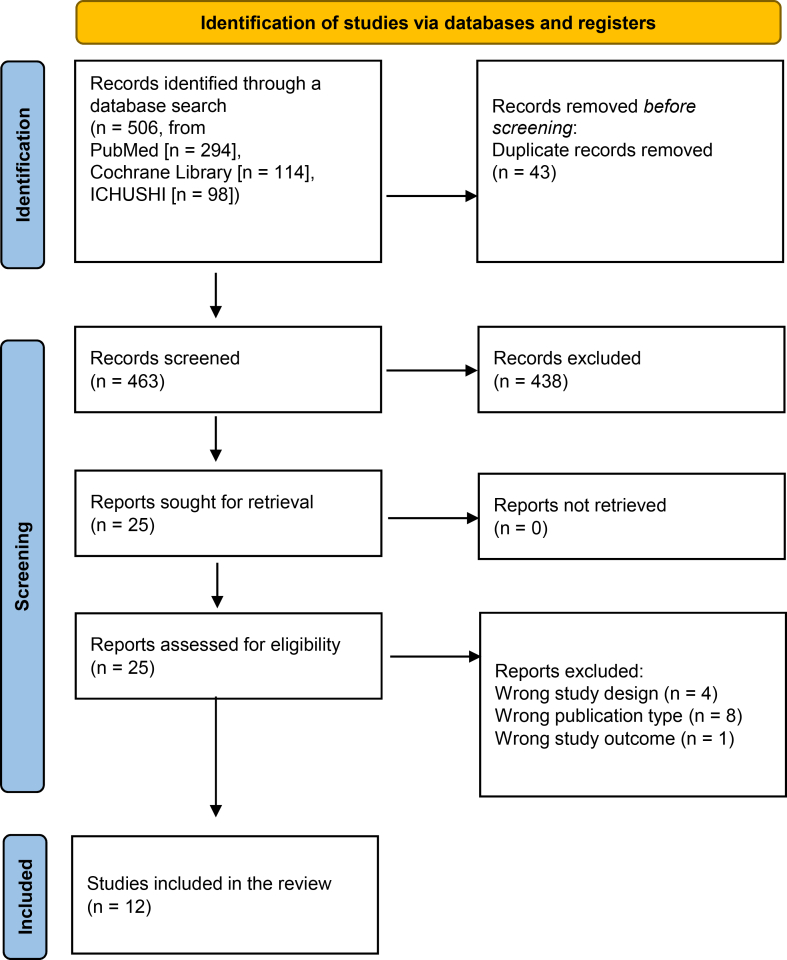
Preferred Reporting Items for Systematic reviews and Meta-Analyses study flow diagram.

Our systematic literature search identified 506 articles, 43 of which were duplicates. After screening the titles and abstracts, 438 articles were excluded because they did not meet the eligibility criteria, leaving 25 articles for full-text review. Of them, 13 were excluded because they did not meet the inclusion criteria. Ultimately, 12 studies involving 289 participants were included in the meta-analysis^23^, ^24^, ^25^, ^26^, ^27^, ^28^, ^29^, ^30^, ^31^, ^32^, ^33^, ^34^ (Fig 1).

### Study characteristics

The characteristics of the 12 studies included in this systematic review are summarized in Table 1. These studies were published between 2012 and 2024. Among these, 6 studies^23^^,^^27^^,^^28^^,^^30^^,^^31^^,^^33^ were published in 2020, whereas the remaining 6^24^, ^25^, ^26^^,^^29^^,^^32^^,^^34^ were published in 2021 or later. Four studies were conducted in Japan,^24^^,^^28^^,^^33^^,^^34^ 4 in Brazil,^25^^,^^26^^,^^30^^,^^31^ 1 in the Czech Republic,^23^ 1 in South Korea,^29^ 1 in Germany,^32^ and 1 in the United Kingdom.^27^ All studies employed an RCT design, including 1 randomized crossover trial.^34^ The sample sizes ranged between 11 and 40 participants. Although this review aimed to include studies involving adults with kidney disease across dialysis and predialysis setting, all participants in the included studies were patients receiving HD. None of the studies involved individuals on peritoneal dialysis, in the predialysis stage, or individuals receiving kidney transplantation. One study specifically included patients undergoing HD with reduced daily physical activity.^24^

**Table 1:** Characteristics of the Included Studies

Study	Country	Participants, n (M/F)	Age	Intervention	Protocol	Outcomes/Measure in this Meta-analysis
Dobsak^23^ (2012)	Czech Republic	Patients undergoing HD IG: 11 (6/5) CG: 10 (4/6)	IG: 64.5 ± 8.1^a^ CG: 60.1 ± 8.2	Intradialytic NMES	IG: Stimulation frequency: 10 Hz; pulse width: 200 ms; rise and fall time: 1 s; on–off mode: 20-s stimulation, 20-s rest; amplitude: 60 mA; applied to quadriceps and calves of both legs. 60 min/session; 3 times/wk for 20 wk CG: No intervention	– Functional capacity (6MWD) – Muscle strength (isometric knee extension strength) – HRQoL (SF-36 physical and mental components summary) – Adverse event (excessive or dangerous fluctuations of hemodynamic parameters)
Homma^24^ (2022)	Japan	Patients undergoing HD with reduced daily physical activity IG: 10 (7/3) CG: 10 (6/4)	IG: 79.4 ± 6.5^a^ CG: 78.4 ± 6.2	Intradialytic belt electrode-skeletal muscle stimulation (B-SES)	IG: Stimulation frequency: 20 Hz; pulse width: 250 μs; on–off mode: 5-s stimulation, 2-s rest; stimulation intensity: maximum tolerated by the patients; applied to lower legs. 40 min/session; 3 times/wk for 12 wk CG: No intervention	– Functional capacity (6MWD) – Physical performance (SPPB) – Body composition (skeletal muscle mass) – ADLs (Functional Independence Measure)
Marini^25^ (2021a)	Brazil	Patients undergoing HD IG: 10 (6/4) CG: 11 (7/4)	IG: 37.3 ± 2.9^a^ CG: 45.8 ± 3.3	Intradialytic NMES	IG: Three-phase NMES protocol: (1) warm-up: 5 min at 5 Hz, 250 μs (continuous mode); (2) muscle stimulation: 30 min at 100 Hz, 400 μs (burst/contraction mode), stimulation intensity: maximum tolerated by the patients; (3) relaxation: 5 min at 5 Hz, 250 μs (continuous mode). Applied bilaterally to the vastus lateralis and gastrocnemius muscles of both legs; 40 min/session; 3 times/wk for 4 wk CG: Sham treatment	– Body composition (appendicular skeletal muscle mass) – Lower-limb muscle strength (one-repetition maximum leg extension test)
Marini^26^ (2021b)	Brazil	Patients undergoing HD IG: 10 (6/4) CG: 11 (7/4)	IG: 37.3 ± 9.2^a^ CG: 45.8 ± 10.8	Intradialytic NMES	IG: Three-phase NMES protocol: (1) warm-up: 5 min at 5 Hz, 250 μs (continuous mode); (2) muscle stimulation: 30 min at 100 Hz, 400 μs (burst/contraction mode), stimulation intensity: maximum tolerated by the patients; (3) relaxation: 5 min at 5 Hz, 250 μs (continuous mode). Applied bilaterally to the vastus lateralis and gastrocnemius muscles of both legs; 40 min/session; 3 times/wk for 4 wk CG: No intervention	– HRQoL (SF-36 physical and mental components summary)
McGregor^27^ (2018)	United Kingdom	Patients undergoing HD IG: 17 (14/3) CG: 18 (11/7)	IG: 51.5 ± 9.1^a^ CG: 54.3 ± 10.2	Intradialytic NMES	IG: Stimulation frequency: 5 Hz; stimulation intensity: adjusted between 5 and 140 mA to the maximum tolerable level. Applied bilaterally to the quadriceps and hamstrings; 60 min/session; 3 times/wk for 10 wk CG: No intervention	– Muscle strength (isometric knee extension strength) – Adverse event (post-exercise hypotension)
Miura^28^ (2018)	Japan	Patients undergoing HD IG: 10 (7/3) CG: 10 (8/2)	IG: 68.6 ± 1.4^b^ CG: 69.9 ± 0.9	Intradialytic NMES	IG: Stimulation frequency: 10 Hz; on–off mode: 20-s stimulation, 20-s rest; stimulation intensity; below pain threshold. Applied bilaterally to the quadriceps and triceps surae muscles; 60 min/session; 2 times/wk for 12 wk CG: No intervention	– Muscle strength (isometric knee extension strength)
Park^29^ (2024)	South Korea	Patients undergoing HD IG: 15 (13/2) CG: 15 (11/4)	IG: 63.0 ± 13.8^a^ CG: 62.0 ± 11.5	Intradialytic NMES	IG: Stimulation frequency: 60 Hz; stimulation intensity: adjusted between 0 and 100 mA to the maximum tolerable level; pulse width: 400 μs; on–off mode: 4-s stimulation, 8-s rest. Applied bilaterally to the vastus medialis and vastus lateralis; 20 min/session in the first week, increased by 2 min/wk up to 30 min; 3 times/wk for 12 wk CG: No intervention	– Muscle strength (isometric knee extension strength) – Body composition (appendicular skeletal muscle mass) – Physical performance (TUG) – Physical activity (International Physical Activity Questionnaire) – HRQoL (KDQOL-SF, physical and mental components summary) – Adverse event (vital signs, laboratory findings)
Roxo^30^ (2016)	Brazil	Patients undergoing HD IG: 20 (9/11) CG: 20 (11/9)	IG: 46.4 ± 15.4^a^ CG: 54.7 ± 19.9	Intradialytic NMES	IG: Stimulation frequency: 50 Hz; stimulation intensity: adjusted to the maximum tolerable level by the patient; pulse width: 350 μs; on–off mode: 2-s stimulation, 10-s rest; applied bilaterally to the quadriceps femoris; 30 min/session; 3 times/wk for 8 wk CG: No intervention	– Lower-limb muscle strength (one-repetition maximum leg extension test) – Functional capacity (6MWD)
Schardong^31^ (2017)	Brazil	Patients undergoing HD IG: 11 (9/2) CG: 10 (8/2)	IG: 59.0 (45.0-72.0)^c^ CG: 64.5 (57.5-67.8)	Intradialytic NMES	IG: Stimulation frequency: 80 Hz; stimulation intensity: adjusted to the maximum tolerable level by the patient; pulse width: 400 μs; on–off mode: 10-s stimulation, rest time decreasing every 2 wk (starting at 50 s, decreasing by 10 s every 2 wk). Applied bilaterally to the quadriceps femoris. Session duration started at 20 min per session and increased by 2 min/wk, reaching 34 min; 3 times/wk for 8 wk CG: No intervention	– Muscle strength (isometric knee extension strength) – Functional capacity (6MWD) – Physical performance (STS-30)
Schinner^32^ (2023)	Germany	Patients undergoing HD IG: 12 (10/2) CG: 11 (8/3)	IG: 66.8 ± 9.8^a^ CG: 72.0 ± 11.4	Intradialytic NMES	IG: Stimulation frequency: 20 Hz; stimulation intensity: adjusted based on the patient’s subjective sensation; pulse width: 250 μs; on–off mode: 5-s stimulation, 2-s rest. Applied bilaterally to the quadriceps femoris 2-3 times/wk, with a total of 60 min/wk for 12 wk CG: No intervention	– Muscle strength (isometric knee extension strength) – Physical performance (STS-60) – Adverse event (leg cramps)
Suzuki^33^ (2018)	Japan	Patients undergoing HD IG: 13 (12/1) CG: 13 (12/1)	IG: 66.2 ± 12.8^a^ CG: 65.1 ± 8.1	Intradialytic NMES	IG: Stimulation frequency: 20 Hz; stimulation intensity: adjusted to the maximum tolerable level by the patient; pulse width: 250 μs; on–off mode: 5-s stimulation, 2-s rest; applied to the gluteal and upper- and lower-leg muscle groups; 20 min/session; 3 times/wk for 8 wk CG: No intervention	– Muscle strength (isometric knee extension strength) – Physical performance (TUG) – HRQoL (SF-36 physical and mental components summary) – Adverse event (leg cramps)
Tsurumi^34^ (2022)	Japan	Patients undergoing HD 11 (7/4)	74.0 ± 5.2^a^	Intradialytic NMES	IG: Stimulation frequency: 4 Hz; stimulation intensity: adjusted to the maximum tolerable level by the patient; pulse width: 250 ms; applied to the lower limbs and abdomen; 30 min/session; 3 times/wk for 12 wk CG: No intervention Crossover design	– Body composition (appendicular skeletal muscle mass) – Physical performance (TUG, SPPB) – Functional capacity (6MWD)

Abbreviations: 6MWD, 6-min walk distance; ADLs, activities of daily living; CG, control group; F, female; HD, hemodialysis; HRQoL, health-related quality of life; IG, intervention group; KDQOL-SF, Kidney Disease Quality of Life Short Form; M, male; NMES, neuromuscular electrical stimulation; SF-36, 36-item Short-Form Health Survey; SPPB, Short Physical Performance Battery; STS-30, 30 s sit-to-stand test; STS-60, 1-min sit-to-stand test; TUG, timed up and go test.

a Mean ± standard deviation.

b Mean ± standard error.

c Median (interquartile range).

In the 12 studies,^23^, ^24^, ^25^, ^26^, ^27^, ^28^, ^29^, ^30^, ^31^, ^32^, ^33^, ^34^ the stimulation frequencies with NMES ranged between 4 and 100 Hz. The pulse durations were 250-400 μs in 8 studies^24^, ^25^, ^26^^,^^29^, ^30^, ^31^, ^32^, ^33^ and 200-250 ms in 2 studies,^23^^,^^34^ whereas 2 studies^27^^,^^28^ did not specify pulse duration. Regarding stimulation intensity, 1 study^23^ applied a fixed amplitude of 60 mA, whereas in the remaining studies,^24^, ^25^, ^26^, ^27^, ^28^, ^29^, ^30^, ^31^, ^32^, ^33^, ^34^ the intensity was adjusted to the maximum tolerable level by the participants.

The intervention frequency varied across the studies, with NMES applied thrice per week in 10 studies,^23^, ^24^, ^25^, ^26^, ^27^^,^^29^, ^30^, ^31^^,^^33^^,^^34^ twice per week in 1 study,^28^ and 2 to 3 times per week in 1 study.^32^ The session duration ranged within 20-60 minutes; the overall study duration ranged within 4-20 weeks. Notably, only 1 study^23^ had an intervention duration >12 weeks, whereas the remaining 11 studies^24^, ^25^, ^26^, ^27^, ^28^, ^29^, ^30^, ^31^, ^32^, ^33^, ^34^ lasted ≤12 weeks. In all studies, the control group received HD and usual care without additional intervention.

### Risk of Bias and Quality of Evidence

**Figure 2:**
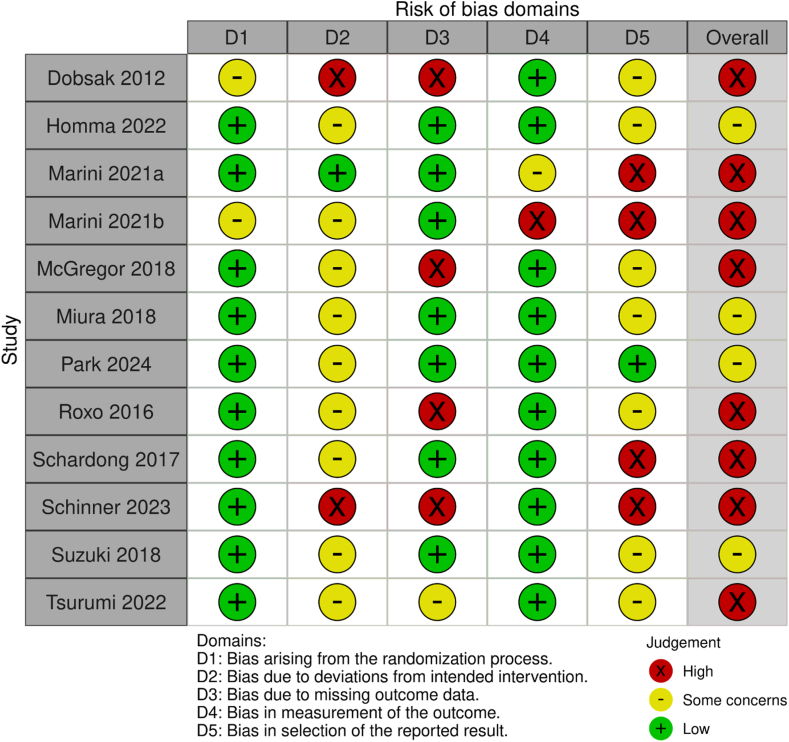
Risk-of-bias summary.

A summary of the risk of bias in the 12 studies is shown in Fig 2. Overall, 8 studies had a high risk of bias,^23^^,^^25^, ^26^, ^27^^,^^30^, ^31^, ^32^^,^^34^ whereas 4 had some concerns.^24^^,^^28^^,^^29^^,^^33^ Among the 8 studies with an overall high risk of bias, 2^23^^,^^32^ had a high risk of bias due to deviations from the intended intervention, 4^23^^,^^27^^,^^30^^,^^32^ due to missing outcome data, 1^26^ due to outcome measurements, and 4^25^^,^^26^^,^^31^^,^^32^ due to the selection of the reported results. One study^34^ did not have a high risk of bias in any specific domain but was classified as having high-risk overall bias due to concerns about multiple domains. Among the 4 studies with some concerns, all four^24^^,^^28^^,^^29^^,^^33^ had some concerns about deviations from the intended intervention, and 3 studies^24^^,^^28^^,^^33^ had some concerns about the selection of the reported results.

The evidence profiles are presented in Table S2.

### Effects of Interventions

#### Lower-Limb Muscle Strength

**Figure 3:**
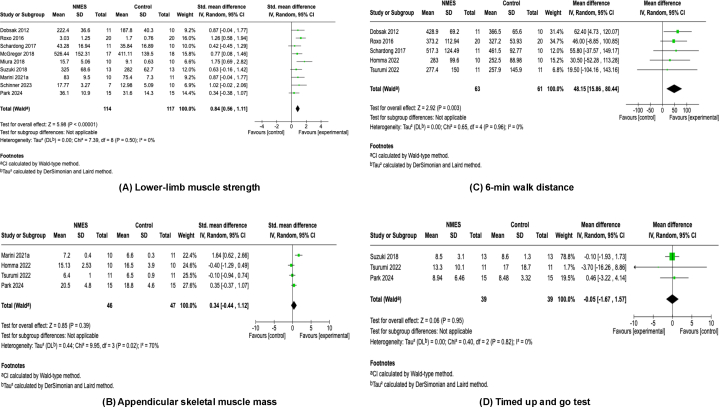

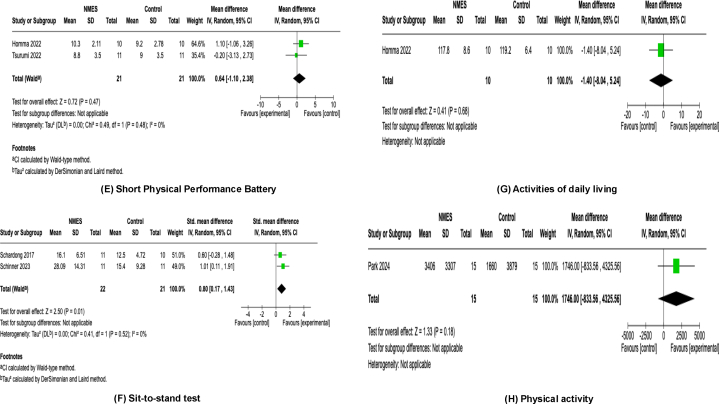

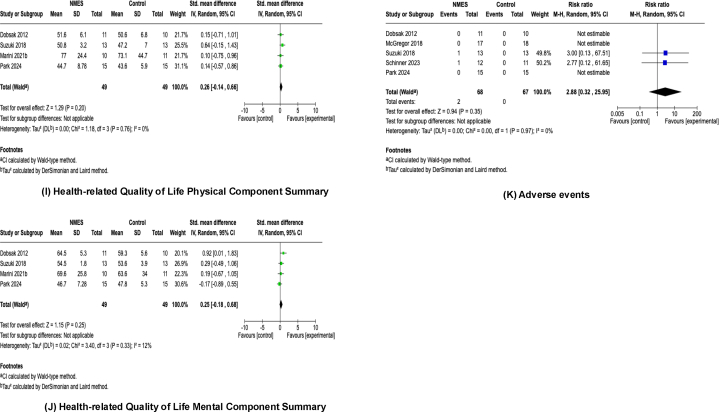
Forest plots of the effects of neuromuscular electrical stimulation. (A) Lower-limb muscle strength. (B) Appendicular skeletal muscle mass. (C) 6-minute walk distance. (D) Timed up and go test. (E) Short Physical Performance Battery. (F) Sit-to-stand test. (G) Activities of daily living. (H) Physical activity. (I) Health-related Quality of Life Physical Component Summary. (J) Health-related Quality of Life Mental Component Summary. (K) Adverse events. CI, confidence interval; IV, inverse variance; NMES, neuromuscular electrical stimulation; SD, standard deviation.

Lower-limb muscle strength was assessed in 9 studies.^23^^,^^25^^,^^27^, ^28^, ^29^, ^30^, ^31^, ^32^, ^33^ Among these, 7 studies^23^^,^^27^, ^28^, ^29^^,^^31^, ^32^, ^33^ evaluated isometric knee extension strength using dynamometry, whereas 2 studies^25^^,^^30^ assessed it by a one-repetition maximum leg extension test. In total, 231 patients (114 in the NMES group, 117 in the control group) from 9 studies were included in the meta-analysis. The NMES intervention significantly enhanced lower-limb muscle strength with a pooled effect size of SMD = 0.84 (95% CI, 0.56-1.11; *P* < 0.001) and low heterogeneity (*I*^2^ = 0%, *P* = 0.50) (Fig 3A).

#### Muscle Mass

Muscle mass, defined as appendicular skeletal muscle mass, was assessed in 4 studies.^24^^,^^25^^,^^29^^,^^34^ Among these, 3 studies^24^^,^^29^^,^^34^ evaluated appendicular skeletal muscle mass by bioimpedance analysis, whereas 1^25^ used dual-energy x-ray absorptiometry. Ninety-three patients (46 in the NMES group, 47 in the control group) from 4 studies were included in the meta-analysis. The NMES intervention did not show a significant improvement in appendicular skeletal muscle mass, with a pooled effect size of SMD = 0.34 (95% CI, −0.44 to 1.12; *P* = 0.39) and moderate-to-high heterogeneity (*I*^2^ = 70%, *P* = 0.02) (Fig 3B).

#### Six-Minute Walk Distance

The 6MWD was assessed in 5 studies.^23^^,^^24^^,^^30^^,^^31^^,^^34^ In total, 124 patients (63 in the NMES group, 61 in the control group) from 5 studies were included in the meta-analysis. The NMES intervention significantly enhanced 6MWD with a pooled effect size of MD = 48.15 (95% CI, 15.86-80.44; *P* = 0.003) and low heterogeneity (*I*^*2*^ = 0%, *P* = 0.96) (Fig 3C).

#### Timed Up and Go

The TUG test was assessed in 3 studies.^29^^,^^33^^,^^34^ Seventy-eight patients (39 in the NMES group, 39 in the control group) from 3 studies were included in the meta-analysis. The NMES intervention did not show significant effects on TUG performance, with a pooled effect size of SMD = −0.05 (95% CI, −1.67 to 1.57; *P* = 0.95) and low heterogeneity (*I*^2^ = 0%, *P* = 0.82) (Fig 3D).

#### Short Physical Performance Battery

SPPB was used in 2 studies^24^^,^^34^ as a test of physical performance. Forty-two patients (21 in the NMES group, 21 in the control group) from 2 studies were included in the meta-analysis. The NMES intervention did not show significant effects on SPPB, with a pooled effect size of MD = 0.64 (95% CI, −1.10 to 2.38; *P* = 0.47) and low heterogeneity (*I*^2^ = 0%, *P* = 0.48) (Fig 3E).

#### Sit-to-Stand

Sit-to-stand (STS) was assessed in 2 studies.^31^^,^^32^ Among these, 1 study^31^ evaluated the 30-s STS test (STS-30) and the other^32^ assessed it using the 1-min STS test (STS-60). Forty-three patients (22 in the NMES group, 21 in the control group) from 2 studies were included in the meta-analysis. The NMES intervention significantly enhanced STS function with a pooled effect size of SMD = 0.80 (95% CI, 0.17-1.43; *P* = 0.01) and low heterogeneity (*I*^2^ = 0%, *P* = 0.52) (Fig 3F).

#### Activities of Daily Living

ADLs were assessed in 1 study.^24^ This study evaluated ADLs using the Functional Independence Measure. Twenty patients (10 in the NMES group, 10 in the control group) from 1 study were included in the meta-analysis. The NMES intervention did not show a significant effect on ADL, with a pooled effect size of MD = −1.40 (95% CI, −8.04 to 5.24; *P* = 0.68), and heterogeneity assessment was not applicable (Fig 3G).

#### Physical Activity

Physical activity was assessed in 1 study.^29^ This study evaluated physical activity by the International Physical Activity Questionnaire. Thirty patients (15 in the NMES group, 15 in the control group) from 1 study were included in the meta-analysis. The NMES intervention did not show a significant effect on physical activity, with a pooled effect size of MD = 1,746.0 (95% CI, −833.56 to 4,325.56; *P* = 0.18), and heterogeneity assessment was not applicable (Fig 3H).

### Health-related Quality of Life

The physical component summary and mental component summary of HRQoL were assessed in 4 studies.^23^^,^^26^^,^^29^^,^^33^ Among these, 1 study^29^ used the Kidney Disease Quality of Life Short Form, whereas 3 studies^23^^,^^26^^,^^33^ assessed HRQoL by the 36-Item Short-Form Health Survey. Ninety-eight patients (49 in the NMES group, 49 in the control group) were included in the meta-analysis. The NMES intervention did not show a significant effect on either the physical component summary (SMD, 0.26; 95% CI, −0.14 to 0.66; *P* = 0.20) (Fig 3I) or mental component summary (SMD, 0.25; 95% CI, −0.18 to 0.68; *P* = 0.25) (Fig 3J). Heterogeneity was low for both outcomes (physical component summary: *I*^2^ = 0%, *P* = 0.76; mental component summary: *I*^2^ = 12%, *P* = 0.33).

### Adverse Events

Adverse events were reported in 5 studies.^23^^,^^27^^,^^29^^,^^32^^,^^33^ The types of adverse events assessed varied among the studies; 1 study^23^ evaluated excessive or dangerous fluctuations in hemodynamic parameters, 1 study^27^ assessed postexercise hypotension, 1 study^29^ monitored vital signs and laboratory findings, and 2 studies^32^^,^^33^ evaluated leg cramps. In total, 2 of the 68 patients in the NMES group experienced adverse events compared with none of the 67 patients in the control group. Meta-analysis revealed no significant difference in the incidence of adverse events between the NMES and control group (risk ratio, 2.88; 95% CI, 0.32-25.95; *P* = 0.35). The heterogeneity was low for this outcome (*I*^2^ = 0%, *P* = 0.97) (Fig 3K).

## Discussion

In this systematic review and meta-analysis, we evaluated the effectiveness of NMES in patients receiving HD by updating previous meta-analysis data. Our findings suggest that intradialytic NMES significantly improves lower-limb muscle strength, functional capacity measured with the 6MWD, and physical performance assessed by the STS without significant increase in adverse events among patients undergoing HD. However, no significant improvements were observed in muscle mass, physical performance, SPPB, ADLs, physical activity, or HRQoL.

Compared with previous meta-analysis results, our updated review confirmed the consistent efficacy of NMES, particularly in enhancing lower-limb muscle strength and functional capacity as assessed by the 6MWD and supported its favorable safety profile. Unlike previous meta-analyses^15^^,^^16^ that included both RCTs and non-RCTs, our updated review was limited to RCTs and incorporated several recent studies published after 2021,^24^, ^25^, ^26^^,^^29^^,^^32^^,^^34^ comprising 50% of the included studies. Specifically, 6 of the 12 included RCTs^23^^,^^27^^,^^28^^,^^30^^,^^31^^,^^33^ overlapped with the prior meta-analyses,^15^^,^^16^ whereas the remaining 6 trials were newly added to the present update. This highlights the substantial update to the evidence base provided by the present review. Nonetheless, our findings regarding improvements in lower-limb muscle strength and functional capacity are consistent with those of previous reviews. However, our study showed significant improvement in physical performance, whereas an earlier review^16^ did not. This difference may be attributed to the type of STS used; the prior analysis included a 10-s STS test (STS-10) in non-RCTs and STS-30, whereas our meta-analysis included STS-30 and STS-60. As the STS-60 requires a longer duration of effort than the STS-10, it is considered to reflect greater muscular endurance and functional capacity and to correlate with the 6MWD scores.^35^^,^^36^ Therefore, NMES may improve muscle strength and functional endurance and capacity, potentially through enhanced muscle oxygenation and fatigue resistance.^37^

Although our study demonstrated significant improvements in muscle strength, no significant changes in muscle mass were observed. This result is consistent with the finding in a previous meta-analysis.^16^ Several factors might explain this lack of effect on appendicular skeletal muscle mass. First, 11 of the 12 included studies had intervention durations of ≤12 weeks. In older adults, resistance training aimed at muscle hypertrophy is typically recommended for at least 8-12 weeks.^38^ However, in patients undergoing dialysis, muscle hypertrophy may require a longer duration because muscle protein synthesis is suppressed by chronic inflammation and the presence of uremic toxins. Therefore, the intervention periods in the studies included in this meta-analysis may have been insufficient to promote increase in muscle mass in patients undergoing dialysis. Second, patients undergoing HD often experience protein-energy wasting due to reduced nutrient intake and loss of amino acids during dialysis.^39^ Therefore, nutritional management in conjunction with NMES might be necessary for promoting muscle hypertrophy. Third, muscle mass measurements by dual-energy x-ray absorptiometry and bioimpedance analysis are affected by the hydration status of patients.^40^ Hence, in patients undergoing HD with volume overload and edema, there is risk of inaccurate muscle mass assessment.

Furthermore, no significant improvements were observed in other outcomes, such as the TUG, SPPB, ADLs, physical activity, and HRQoL. The TUG, SPPB, and ADL assessments reflect comprehensive physical performance and thus may not be sufficiently improved by NMES alone. Moreover, in the case of HRQoL, the passive nature of the NMES intervention might have limited the enhancement of self-efficacy. Moreover, physical activity is associated with mortality risk in patients,^41^^,^^42^ highlighting the importance of increasing habitual physical activity. Therefore, strategies that encourage patients to increase their habitual physical activity, including monitoring using wearable devices, may be useful.^43^ These findings suggest that in patients undergoing HD, a comprehensive approach combining NMES with physical exercise training, ADL-based exercises, and strategies for promoting physical activity might be more effective than NMES alone.

### Limitations

This systematic review and meta-analysis had some limitations. First, most included studies had relatively small sample sizes, which might have limited the statistical power to detect meaningful differences, particularly in outcomes. In addition, since fewer than 10 studies were available for each outcome, we could not formally assess publication bias (eg, using funnel plots or Egger’s/Begg’s tests^22^); therefore, publication bias cannot be excluded. Second, the methodological quality of the included trials was generally low; based on the RoB 2.0 assessment, 8 of 12 studies were rated as having a high risk of bias and the remaining 4 as having some concerns, with none judged as low risk. Additionally, the certainty of evidence was rated as low or very low for most outcomes according to the GRADE system, including those that showed statistically significant effects of NMES. To improve the quality of evidence, future trials should use more robust methodological designs to validate the efficacy of NMES in patients undergoing HD. Third, although in this systematic review, we aimed to broadly assess patients at various stages of CKD, including patients with predialysis CKD, those undergoing peritoneal dialysis, and post-kidney transplant recipients, all included studies were limited to patients undergoing HD. Consequently, the effects of NMES in patients with predialysis CKD or peritoneal dialysis or kidney transplant recipients could not be evaluated. Given that patients with predialysis CKD, those on peritoneal dialysis, and kidney transplant recipients also experience declines in physical function and sarcopenia, similar to those undergoing HD,^5^ exploring the broader applicability of NMES across different kidney disease populations is important. Moreover, unlike in-center HD settings, the timing and implementation of NMES in these populations might require tailored strategies for ensuring its feasibility and effectiveness. Fourth, the NMES protocols substantially varied across the studies in terms of stimulation frequency, intensity, duration, and total intervention period. This heterogeneity might have affected the results, making identifying the optimal NMES settings in this population difficult.

### Implications

As a passive modality that can be implemented during dialysis sessions, NMES holds practical value to patients who are unable or unwilling to engage in conventional exercises. Integration into intradialytic exercise programs might offer a feasible strategy for maintaining or enhancing physical function in vulnerable populations.

### Conclusions

This systematic review and meta-analysis provides an updated and methodologically robust evaluation of the effects of NMES on patients undergoing HD. Although this review was initially intended to include a broad range of kidney disease populations, all included studies were limited to those receiving HD. Our findings demonstrated that NMES significantly improved lower-limb muscle strength, functional capacity (6MWD), and physical performance (STS), without increasing the risk of adverse events. These results strengthen the current evidence and support the integration of NMES as a safe and effective component of intradialytic rehabilitation programs. To further validate its utility, future high-quality trials with standardized protocols and expanded populations are warranted.
