# Supplementary material for: Efficacy of Neuromuscular Electrical Stimulation in Patients on Hemodialysis: An Updated Systematic Review and Meta-analysis
Source: Kidney Med. 2026 Apr 16;8(6):101368. doi: 10.1016/j.xkme.2026.101368 (PMC13218137; doi:10.1016/j.xkme.2026.101368)
Supplement: Supplementary File (PDF) — Table S1-S2. [file mmc1.pdf]

Table S1.

Search strategy

Chart 1: PubMed search strategy

(("Kidney Diseases" [mesh] OR "renal disease*" [tiab] OR "kidney disease*" [tiab] OR "kidney disorder*" [tiab] OR "kidney failure*" [tiab] OR "renal failure*" [tiab] OR "renal insufficienc*" [tiab] OR "kidney insufficienc*" [tiab] OR "CKD" [tiab] OR "CKF" [tiab] OR "ESKD" [tiab] OR "ESKF" [tiab] OR "ESRF" [tiab] OR "ESRD" [tiab] OR "Renal Replacement Therapy" [mesh] OR "Renal Replacement Therap*" [tiab] OR "RRT" [tiab] OR "hemodialysis" [tiab] OR "haemodialysis" [tiab] OR "hemofiltration*" [tiab] OR "haemofiltration*" [tiab] OR "dialysis" [tiab] OR "dialized" [tiab] OR "Peritoneal Dialysis" [tiab] OR "HDF" [tiab] OR "renal transplant*" [tiab] OR "renal recipient*" [tiab] OR "kidney transplant*" [tiab] OR "kidney recipient*" [tiab]) AND ("NMES" [tiab] OR "Electrical Muscle Stimulation*" [tiab] OR "Electric Stimulation*" [mesh] OR "Electric Stimulation Therapy" [mesh:noexp] OR "Electric Stimulation*" [tiab] OR "Electrical Stimulation*" [tiab])) NOT ("Animals"[mesh:noexp])

Chart 2: Cochran Library search strategy

([mh "Kidney Diseases"] OR (renal NEXT disease*):ti,ab,kw OR (kidney NEXT disease*):ti,ab,kw OR (kidney NEXT disorder*):ti,ab,kw OR (kidney NEXT failure*):ti,ab,kw OR (renal NEXT failure*):ti,ab,kw OR (renal NEXT insufficienc*):ti,ab,kw OR (kidney NEXT insufficienc*):ti,ab,kw OR (CKD):ti,ab,kw OR (CKF):ti,ab,kw OR (ESKD):ti,ab,kw OR (ESKF):ti,ab,kw OR (ESRF):ti,ab,kw OR (ESRD):ti,ab,kw OR [mh "Renal Replacement Therapy"] OR (Renal NEXT Replacement NEXT Therap*):ti,ab,kw OR (RRT):ti,ab,kw OR (hemodialysis):ti,ab,kw OR (haemodialysis):ti,ab,kw OR (hemofiltration*):ti,ab,kw OR (haemofiltration*):ti,ab,kw OR (dialysis):ti,ab,kw OR (dialized):ti,ab,kw OR (Peritoneal NEXT Dialysis):ti,ab,kw OR (HDF):ti,ab,kw OR (renal NEXT transplant*):ti,ab,kw OR (renal NEXT recipient*):ti,ab,kw OR (kidney NEXT transplant*):ti,ab,kw OR (kidney NEXT recipient*):ti,ab,kw) AND ((NMES):ti,ab,kw OR (Electrical NEXT Muscle NEXT Stimulation*):ti,ab,kw OR (Electric NEXT Stimulation*):ti,ab,kw OR [mh ^"Electric Stimulation Therapy"] OR (Electric NEXT Stimulation*):ti,ab,kw OR (Electrical NEXT Stimulation*):ti,ab,kw)

Chart 3:“ICHUSHI”search strategy

((腎臓疾患/TH or 腎臓疾患/TA or 腎疾患/TA or 腎症/TA or 腎臓病/TA or 腎機能不全/TA or 腎機能障害/TA or 腎機能低下/TA or 腎不全/TA or 慢性腎臓病/TA or 腎代替療法/TH or 腎代替療法/TA or 腎置換療法/TA or 透析/TA or 腎移植/TA or 腎臓移植/TA) and (神経筋電気刺激/TA or NMES/TA or 電気的筋肉刺激/TA or EMS/TA or 電気刺激/TH or @"電気刺激療法"/TH or 電気刺激/TA or 電気刺激療法/TA)) and (LA=日本語) not ((PT=症例報告) or (PT=会議録))not (CK=動物)

Table S2.

Quality of evidence evaluated through the Grading of Recommendations, Assessment, Development, and Evaluation (GRADE)system

Certainty assessment							№ of patients		Effect		Certainty	Importance
№ of studies	Study design	Risk of bias	Inconsistency	Indirectness	Imprecision	Other considerations	NMES	Control	Relative (95% CI)	Absolute (95% CI)		
**Lower-limb muscle strength**												
9	randomised trials	serious[a]	serious[b]	not serious	not serious	none	114	117	-	SMD **0.84 higher** (0.56 higher to 1.11 higher)	⨁⨁◯◯ Low[a,b]	CRITICAL
**Appendicular Skeletal Muscle Mass**												
4	randomised trials	serious[a]	serious[b]	not serious	not serious	none	46	47	-	SMD **0.34 higher** (0.44 lower to 1.12 higher)	⨁⨁◯◯ Low[a,b]	CRITICAL
**6-minute walk distance**												

Certainty assessment							№ of patients		Effect		Certainty	Importance
№ of studies	Study design	Risk of bias	Inconsistency	Indirectness	Imprecision	Other considerations	NMES	Control	Relative (95% CI)	Absolute (95% CI)		
5	randomised trials	very serious[a]	serious[c]	not serious	not serious	none	63	61	-	MD **48.15 higher** (15.86 higher to 80.44 higher)	⊕◯◯◯ Very low[a,c]	CRITICAL
**Timed-Up and Go Test**												
3	randomised trials	not serious	serious[b]	not serious	not serious	none	39	39	-	MD **0.05 lower** (1.67 lower to 1.57 higher)	⊕⊕⊕◯ Moderate[b]	CRITICAL
**Short Physical Performance Battery**												

Certainty assessment							№ of patients		Effect		Certainty	Importance
№ of studies	Study design	Risk of bias	Inconsistency	Indirectness	Imprecision	Other considerations	NMES	Control	Relative (95% CI)	Absolute (95% CI)		
2	randomised trials	serious[a]	serious[c]	not serious	not serious	none	21	21	-	MD **0.64 higher** (1.1 lower to 2.38 higher)	⊕⊕◯◯ Low[a,c]	CRITICAL
**Sit-and-Stand Test**												
2	randomised trials	very serious[a]	serious[b]	not serious	not serious	none	22	21	-	SMD **0.8 higher** (0.17 higher to 1.43 higher)	⊕◯◯◯ Very low[a,b]	CRITICAL

Functional Independence Measure

Certainty assessment							№ of patients		Effect		Certainty	Importance
№ of studies	Study design	Risk of bias	Inconsistency	Indirectness	Imprecision	Other considerations	NMES	Control	Relative (95% CI)	Absolute (95% CI)		
1	randomised trials	not serious	serious[b]	not serious	not serious	none	10	10	-	MD **1.4 lower** (8.04 lower to 5.24 higher)	⨁⨁⨁◯ Moderate[b]	CRITICAL

Physical Activity

1	randomised trials	not serious	serious[c]	not serious	not serious	none	15	15	-	MD **1746 higher** (833.56 lower to 4325.56 higher)	⨁⨁⨁◯ Moderate[c]	CRITICAL

Health-related Quality of Life Physical Component Summary

Certainty assessment							№ of patients		Effect		Certainty	Importance
№ of studies	Study design	Risk of bias	Inconsistency	Indirectness	Imprecision	Other considerations	NMES	Control	Relative (95% CI)	Absolute (95% CI)		
4	randomised trials	serious[a]	serious[b]	not serious	not serious	none	49	49	-	SMD **0.26 higher** (0.14 lower to 0.66 higher)	⊕⊕◯◯ Low[a,b]	CRITICAL
**Health-related Quality of Life Mental Component Summary**												
4	randomised trials	serious[a]	serious[b]	not serious	not serious	none	49	49	-	SMD **0.25 higher** (0.18 lower to 0.68 higher)	⊕⊕◯◯ Low[a,b]	CRITICAL
**New Pain Onset**												

Certainty assessment							№ of patients		Effect		Certainty	Importance
№ of studies	Study design	Risk of bias	Inconsistency	Indirectness	Imprecision	Other considerations	NMES	Control	Relative (95% CI)	Absolute (95% CI)		
1	randomised trials	not serious	very serious[c]	not serious	not serious	none	3/13 (23.1%)	0/13 (0.0%)	**RR 7.00** (0.40 to 123.35)	**0 fewer per 1,000** (from 0 fewer to 0 fewer)	⨁⨁◯◯ Low[c]	CRITICAL
**Adverse Event**												
5	randomised trials	serious[a]	very serious[c]	not serious	not serious	none	2/68 (2.9%)	0/67 (0.0%)	**RR 2.88** (0.32 to 25.95)	**0 fewer per 1,000** (from 0 fewer to 0 fewer)	⨁◯◯◯ Very low[a,c]	CRITICAL

CI: confidence interval; **MD:** mean difference; **RR:** risk ratio; **SMD:** standardized mean difference

Explanations

a. Multiple high-risk biases

b. Small sample size

c. Wide confidence interval
